# Circulating Tumor Cells: From the Laboratory to the Cancer Clinic

**DOI:** 10.3390/cancers12092361

**Published:** 2020-08-21

**Authors:** Ruchi Agashe, Razelle Kurzrock

**Affiliations:** Center for Personalized Cancer Therapy, University of California San Diego, Moores Cancer Center, La Jolla, CA 92130, USA; ruchi.agashe@yale.edu

**Keywords:** circulating tumor cells, ctDNA, cancer

## Abstract

Circulating tumor cells (CTCs) are cells that are shed from tumors into the bloodstream. Cell enrichment and isolation technology as well as molecular profiling via next-generation sequencing have allowed for a greater understanding of tumor cancer biology via the interrogation of CTCs. CTC detection can be used to predict cancer relapse, progression, and survival; evaluate treatment effectiveness; and explore the ex vivo functional impact of agents. Detection methods can be by either immunoaffinity (positive or negative enrichment strategies) or biophysical strategies. CTC characterization, which is performed by DNA, RNA, and/or protein techniques, can predict metastatic potential. Currently, CTC-derived explant models may mimic patient response to chemotherapy and help with studying druggable targets and testing treatments. The Food and Drug Administration has cleared a CTC blood test to enumerate CTCs derived from breast, prostate, and colorectal cancers. In conclusion, liquid biopsies via CTCs provide a non-invasive way to obtain important diagnostic, prognostic, and predictive information in patients with cancer.

## 1. Introduction

Circulating tumor cells, or CTCs, are cells that are shed from tumors into the bloodstream. After release from tumors, most CTCs die in the circulation (in ~1–2.5 h) owing to mechanical forces or immune system attack [1]. However, a small fraction of CTCs survive and seed distant metastatic disease.

Technology has now allowed for the detection of CTCs and their interrogation at the molecular and functional levels [2]. The emerging methods of cell enrichment, isolation, and analysis have given rise to a greater understanding of tumor cancer biology [3]. Molecular profiling of the cells using next-generation sequencing (NGS) allows for a comprehensive analysis of genomic alterations in CTCs [4]. Additionally, the phenotypic and functional characteristics of the cells may provide information about which types of treatments patients should receive, and CTC enumeration and characterization may also be important for determining prognosis [5].

The average patient with metastatic cancer has between 5 and 50 CTCs for about every 7.5 mL of blood [6]. Hence, CTCs occur at extremely low levels in the circulation and are obscured by billions of peripheral blood cells. This small number restricts the utility of CTCs. Still, CTCs provide reliable “omic” information, and their enumeration is also of clinical importance. Indeed, numerous studies have reported that CTC detection as a “liquid biopsy” can be used as a marker to predict cancer relapse, progression, and survival [7,8].

CTCs hold keys to understanding metastasis [7]. High CTC numbers correlate with high tumor burden, aggressive disease, and shorter time to relapse. CTCs also demonstrate significant potential in therapeutic management, from simple enumeration of CTCs for the evaluation of treatment effectiveness to molecular analysis for choosing targeted therapies, and CTC culture informs resistance mechanisms. In addition, by exploiting ultrasensitive molecular platforms, CTCs may significantly increase the ability to diagnose early-stage cancer. Finally, the comprehensive molecular characterization of CTCs using advanced “omic” technologies amplifies our understanding of the biology of cancer.

Compared with other forms of liquid biopsy, such as circulating tumor DNA/RNA (ctDNA/RNA) and extracellular vesicles derived from live tumor cells, CTCs are whole cells with unique morphological information and have distinct advantages for evaluating genomics, transcriptomics, and proteomic signaling. Taken together, enriching CTCs from peripheral blood as a non-invasive substitute for conventional tissue biopsy permits serial, real-time interrogation of the dynamic evolution of neoplasms, holds promise for advancing precision cancer medicine, and enables fundamental research into the biology of malignancies [8].

## 2. Technological Methods to Detect CTCs

There are many different techniques used for CTC enrichment (the ability to capture tumor cells amongst other cells in circulation) and CTC detection (Table 1). These include positive and negative immunoaffinity strategies, as well as biophysical enrichment strategies based on the differential size of CTCs. Positive enrichment captures CTCs by targeting specific antigens. Negative enrichment captures background cells by targeting antigens not expressed by CTCs. Negative enrichment typically has less purity than positive enrichment. Purity refers to the ability to detect CTCs in the presence of contaminating background cells.

### 2.1. CTC Enrichment by Immunoaffinity

CTC enrichment by immunoaffinity is one of the most widely used techniques for CTC capture. Using specific biomarkers (such as EpCAM) expressed on the cell surface, the cells are captured and the antibodies are secured to either a magnetic substance or the device surface. One limitation, however, is that there is no universal CTC-specific antigen. Rather, there are a variety of surface markers that are expressed by CTCs [9].

### 2.2. Positive Enrichment Strategies

Amongst positive enrichment strategies, the CellSearch system is used to monitor patients with metastatic breast, prostate, and colorectal cancers. The system utilizes ferrofluid nanoparticles, which are used to separate epithelial cell adhesion molecule (EpCAM) cells from other blood components after centrifugation [10]. AdnaTest is another technology used for CTC detection. It employs antibody-coated beads that are specific to the type of cancer. After collection, a real-time polymerase chain reaction (RT-qPCR) is run to determine expression patterns [11]. Magnetic-activated cell sorting (MACS) is an enrichment technology based on immunomagnetic separation. It captures cells with magnetic nanoparticles conjugated with antibodies [12,13]. There are also microfluidic-based positive enrichment technologies, and also use of anti-EpCAM antibodies to optimize cell attachment to the antibody-coated posts through the geometric arrangement of microposts [9]. Another micropost-based device is the Target Selector™ CTC platform (Biocept), which uses an antibody cocktail to capture CTCs. This setup is different from typical anti-EpCAM-only-based systems because in addition to antibodies against EpCAM, the capture cocktail includes antibodies that target other mesenchymal and stem cell tumor-associated and cell-type-specific markers. Additionally, the versatility of this system allows for the assessment of various biomarkers at the protein (immunocytochemistry) and DNA levels (FISH) within the microfluidic channels [14].

### 2.3. Negative Enrichment Strategies

There are two main types of negative enrichment technologies used to isolate CTCs: the EasySep system and quadrupole magnetic separation (QMS). The EasySep system uses a similar magnetic technology by incubating samples with magnetic nanoparticles and antibodies targeting CD45 cells. QMS uses a cylindrical separation system that detects immunomagnetically labeled cells [11].

### 2.4. CTC Enrichment Based on Biophysical Properties

Some technologies for CTC separation are based on purely biophysical properties. For example, the size of the tumor cell can play a role because CTCs are generally larger than background cells [15]. Additionally, centrifugation, microfiltration, and dielectrophoresis are used [16].

## 3. Mechanisms by Which Circulating Tumor Cells Enter, Leave, and Travel in the Circulation

**Figure 1:**
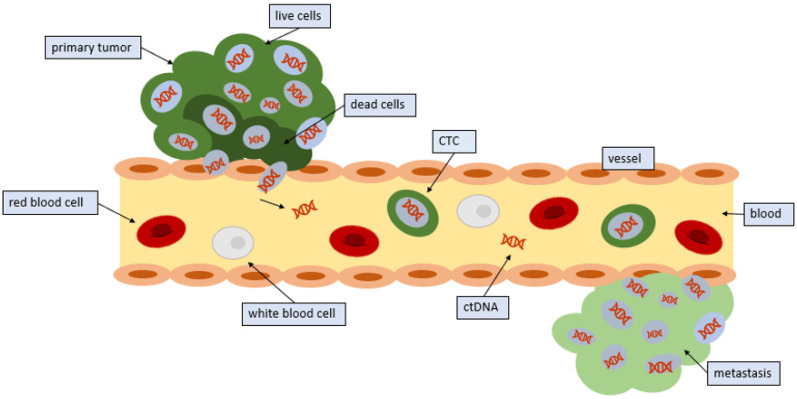
Entrance of CTCs into the circulation: Tumors release CTCs into the circulation. CTCs from tumors often die in the circulation; however, some CTCs survive and seed distant sites.

Endothelial cells are responsible for lining every blood vessel in the body. These cells form an interface called the endothelium, which lets substances in and out of the tumor. Under normal circumstances, the endothelium would not let a cancer cell through (Figure 1). Indeed, CTCs have a diameter that is three to four times as large as the bores of capillaries in distant organs. Therefore, CTCs detected in the blood imply that only small or malleable CTCs can circulate, or that the cancer cells can use signaling to control endothelial cells, thus facilitating the release of CTCs into the circulation [17]. The tumor cells utilize microRNAs, which are strands of RNA that can interfere with messenger RNA, thereby preventing the translation of proteins. With microRNAs, the cell begins to express proteins on their surface that facilitate the adherence of other cells [18]. CTCs are then able to bind to this site and penetrate the vessel. Cytokines in the microenvironment may also influence this process.

Not all CTCs that are shed remain in the circulation. Many CTCs are found in blood near the site of the primary tumor as well as in peripheral blood [19]. CTCs that remain in the circulation typically only remain there for a few hours [1].

Protein–protein interactions and signaling events are the underlying mechanisms of cell migration and play a dominant role in focal adhesion. In order to migrate, the cell body must change its shape and rigidity to interact with the surrounding tissue structures [19]. The cells must bypass the extracellular matrix, which provides a barrier to these cell bodies. Various cell protrusions can initiate extracellular matrix recognition and binding. These protrusions, or pseudopods, are activated by actin polymerization, and elongate until they come into contact with the adjacent extracellular matrix to initiate binding through adhesion molecules. Currently, there are cancer therapeutic strategies aimed at targeting these adhesion molecules to prevent the onset of metastasis [20].

## 4. Characterization of Circulating Tumor Cells by Genomic, RNA, and Protein Techniques

Predicting metastasis from the gene expression profiles of the primary tumor might facilitate an understanding of the shared traits that predispose diverse tumors to metastasize [21,22]. Specific protein signatures may also indicate greater metastatic potential. For protein analysis, immunostaining with antibodies against the protein of interest is typically performed. Further development of this method is ongoing and could lead to information on the identification of signaling pathways relevant to the development of metastasis [5].

In order to identify aberrations in CTCs, one can utilize either DNA, RNA, or protein techniques. Through immunostaining, CTCs are identified and isolated. DNA can be amplified and analyzed for alterations [23]. Additionally, fluorescence in situ hybridization (FISH) techniques can be utilized to identify gene amplifications or gene translocations within individual CTCs [24,25]. Transcriptome (RNA) profiling can be performed by sequencing or by in situ hybridization, which localizes a specific RNA sequence in circulating tumor cells [26].

## 5. CTC-Derived Explant Models

Due to the development of chemotherapy resistance and progression of disease, progression-free survival (PFS) in patients with metastatic disease is often short. Therefore, it is important to understand the biology behind drug resistance, which can be done by using cell cultures that reflect functionality. The study of these advanced and progressive diseases is sometimes limited due to a lack of suitable pathologic tissue [27]. However, CTCs can be exploited to provide more information. A CTC-derived explant (CDX) model in immunocompromised mice has been developed. These CDX models may mimic patient responses to standard chemotherapy, and can be used to study new druggable targets and test current treatments [28]. Additionally, CDX models can assist with the discovery of the mechanism of treatment resistance and tumor cell dissemination [29].

## 6. Clinical Uses of Circulating Tumor Cells

Currently, CTCs are used to determine prognosis in metastatic cancer patients and, by monitoring tumor genotypes, may serve as a prognostic biomarker for clinicians (Table 2). For instance, the CellSearch CTC test is a blood test that has been used to enumerate CTCs derived from numerous cancer types, and is FDA-cleared for breast, prostate, and colorectal cancers [5]. It provides an early assessment of patient prognosis, discerns changes in prognosis with time, and accurately assesses prognosis in cases where the CT and imaging of tumor markers are discordant in patients with metastatic breast or prostate cancer; it is also approved for monitoring patients with metastatic breast, prostate, and colorectal cancers, all of which have decreased progression-free and overall survival if CTCs are detected in the blood. Studies demonstrating significant correlations between CTC counts and metastatic relapse have also been reported in other malignant entities, such as colorectal cancer, bladder cancer, liver cancer, and esophageal cancer [30,31]. CTCs can also be used to identify therapeutic targets and resistance pathways. Studies may be performed at the DNA, RNA, or protein level [32]. Therapeutic targets identified on CTCs include but are not limited to estrogen receptor (ER), human epidermal growth factor receptor 2 (HER2), epidermal growth factor receptor (EGFR), hepatocyte growth factor receptor (MET), KRAS, programmed death-ligand 1 (PD-L1), progesterone receptor (PR), anaplastic lymphoma kinase (ALK), and ROS [33,34,35,36,37].

Studies on prostate cancer have shown that the mRNA analysis of CTCs can provide information about drug resistance. This is important because most patients with hormone-dependent prostate cancer develop resistance to treatment. One example is castration-resistant prostate cancer [46]. Patients with this type of cancer have disease progression despite going through antiandrogen treatment. However, researchers may be able to detect the failure of antiandrogen treatments with enzalutamide and abiraterone by determining the mRNA expression of AR-V7, an active androgen receptor that does not have a ligand-binding domain [47].

## 7. Comparison of CTCs and Circulating Tumor DNA (ctDNA)

Both CTCs and ctDNA are emerging as potential non-invasive biomarkers obtainable via a blood test, also known as a liquid biopsy [48].

Circulating tumor DNA refers to the fragments of DNA that are shed into the bloodstream by the tumor. They can provide information about the tumor genome without the need to isolate CTCs [49]. Due to its highly specific and highly sensitive nature, ctDNA is very informative. It also allows for the monitoring of tumors. The major advantage of ctDNA is that it is easier to isolate than CTCs (Table 3). Currently, the analysis of ctDNA to detect alterations and/or responses to targeted treatment has been performed for colorectal cancer; breast malignancies; esophageal, gastroesophageal junction, and gastric adenocarcinoma; peritoneal carcinomatosis; and more [50,51,52,53].

In contrast, CTCs can be used for DNA analyses and to provide information gleaned from their protein and RNA content. Some of this information is unobtainable from ctDNA. Furthermore, functional assays can be performed on CTCs that are cultured [54]. Additionally, while CTCs can be cultured both in vivo and in vitro, ctDNA cannot be cultured at all. Both can be used to determine response to therapy and predict resistance [55].

## 8. Other Circulating Vehicles: Exosomes

Exosomes are small (30–150 nm) lipid-bilayered vesicles that are released by live cells into the extracellular environment via exocytosis during the fusion of multivesicular bodies with the plasma membrane. They have been identified as a promising biomarker in multiple diseases, and are discharged in various biological fluids (plasma, urine, saliva, etc.) [56]. They often contain important cargo, such as nucleic acids, including mRNA and miRNA. Exosome-based liquid biopsies are advantageous for a variety of reasons: exosomes are more homogenous in terms of size as compared with other subcellular particles, isolation and characterization technologies are already well developed in research, they exhibit specific markers that can be used for separation from other subcellular particles, they are stable in the circulation and found in almost all body fluids, and RNA is often stable and protected from degradation [55,57]. Many studies have shown that exosomes play roles in tumor initiation, progression, metastasis, and drug resistance. Enrichment methods for exosomes are similar to those for CTCs. After enrichment, RT-qPCR, Western blot, fluorescence in situ hybridization (FISH), flow cytometry, and next-generation sequencing (NGS) are used for analysis [58].

## 9. Conclusions and Future Directions

There are still many limitations of CTC technology when it comes to specificity, sensitivity, and reproducibility [59]. More sensitive technologies need to be developed in order to increase the quantity of CTCs that are detected. For instance, while the CellSearch technology has been useful, it relies on high levels of EpCAM expression on the CTCs. Therefore, methods still need to be developed to detect tumor cells with downregulated epithelial proteins [60].

Other issues relate to the findings when the molecular characteristics of CTCs are probed. CTCs may be shed from different locations within tumors (which are heterogeneous in nature), and even from metastases. Indeed, there is often a discrepancy in gene expression between primary tumors and CTCs, as well as heterogeneity within the CTC population [61].

Interestingly, traits in some malignancies have been traced to so-called cancer stem cells (CSCs). The features that define CSCs—self-renewal, tumor-initiating, invasive, motile, and heightened resistance to apoptosis—are also instrumental for metastasis, suggesting that CTCs with high metastatic potential might be CSCs.

Taken together, CTCs are at the frontier of liquid biopsies. The analysis of CTCs has clinical value at multiple levels, including but not limited to the early detection and treatment of metastatic spread and providing critical DNA, RNA, protein, and functional information that enables targeting metastases.

## Figures and Tables

**Table 1:** Detection, Enrichment, and Separation Techniques for CTCs [9].

Technique	Advantages	Limitations
**CellSearch**	Ferrofluid nanoparticles with EpCAM antibodies	Dependent on EpCAM
**AdnaTest**	Immunomagnetic detection of EpCAM, utilizes RT-qPCR for protein expression	Dependent on EpCAM
**ISET**	Size of CTC, immunodetection on EpCAM	Dependent on EpCAM, size variability
**EPISPOT**	Enrichment through antigen expression, enzymatic activity	Variability of enzymatic activity
**FISH**	Detection of chromosomal DNA sequence	Requires cell permeabilization fluorescence probe, time-consuming/labor intensive
**FAST**	Detection through antigen expression	Half of CTCs are lost in the process, highly sensitive to EpCAM marker down-regulation
**Density Gradient**	Enrichment based on cell size, efficient, inexpensive, not dependent on cell surface marker expression	Low specificity, high contamination, CTC loss
**Microfiltration (3D)**	Efficient, captures live CTCs	Costly, dilution of blood required
**Microflow**	Efficient CTC enrichment, undiluted blood	Prototype, false positives and negative results, based on presence of antigens on CTC surface

**Abbreviations**: CTC—circulating tumor cell; EpCAM—epithelial cellular adhesion molecule; EPISPOT—enzyme-linked immunosorbent spot assay; FAST—fiber-optic array scanning technology; FISH—fluorescence-assisted in situ hybridization; ISET—isolation by size of epithelial tumor cells.

**Table 2:** Examples of the clinical utility of CTC detection [1].

Cancer Type	Setting	Results
**Breast cancer**	In a prospective multicenter study, 177 patients with measurable metastatic breast cancer were tested for CTCs before starting new treatment and at first follow up.	The number of circulating CTCs before treatment was an independent predictor of PFS and OS in patients with metastatic breast cancer [38]
**Breast cancer**	Nonmetastatic 91 primary (non-metastatic) breast cancer patients given adjuvant treatment	CTCs are influenced by adjuvant chemotherapy and an increase (even after initial response to therapy) of ≥10-fold at the end of therapy is a strong predictor of relapse and a surrogate marker for the [39]
**Breast cancer**	17 centers provided data for 1944 eligible patients with metastatic breast cancer from 20 studies.	Aggressiveness of the tumor cells. Independent prognostic effect of CTC count on progression-free survival and overall survival [40]
**Non-small-cell lung carcinoma**	41 patients on a phase II clinical trial of erlotinib and pertuzumab.	Correlation between decreases in CTCs and radiographic response in patients with advanced lung cancer [41]
**Non-small-cell lung carcinoma**	87 patients with lung cancer showing CTCs were screened ALK status in both tumor tissue and in CTCs	ALK status can be determined in CTCs isolated from patients with lung cancer by immunocytochemistry and FISH analyses [42]
**Hepatocellular carcinoma (HCC)**	CTCs were detected by a novel method. Prevalence of CTCs was examined in samples from HCC patients, healthy volunteers, and patients with benign liver diseases or non-HCC cancers.	No healthy, benign liver disease or non-HCC subjects had CTCs detected. CTCs were identified in 69 of 85 (81%) HCC patients, with an average of 19 ± 24 CTCs per 5 mL Both the positivity rate and the number of CTCs were significantly associated with tumor size, portal vein tumor thrombus, differentiation status, and the disease extent as classified by TNM (tumor-node-metastasis). *ERBB2* gene amplification and *TP53* gene deletion were detected in CTCs [43]
**Prostate carcinoma**	Prospectively enrolled patients with metastatic, castration-resistant prostate cancer initiating taxane chemotherapy	Detection of AR-V7 in CTCs from men with metastatic castration resistant prostate cancer was not associated with primary resistance to taxane chemotherapy. In AR-V7-positive men, taxanes were more efficacious than androgen blockers enzalutamide or abiraterone therapy, whereas in AR-V7-negative men, taxanes and enzalutamide or abiraterone had comparable efficacy [44]
**Melanoma**	Phase III trial of adjuvant immunotherapy after complete resection of stage Iv melanoma	Pretreatment CTCs (>0 vs. O) status was associated with shorter disease free and overall survival. Serial CTCs was also associated with disease free survival [45]

**Abbreviations**: ALK—anaplastic lymphoma kinase; AR-V7—androgen-receptor splice variant 7; CTC—circulating tumor cell; FISH—fluorescence-assisted in situ hybridization; HCC-hepatocellular cancer; OS—overall survival; PFS—progression-free survival; TNM—tumor, node, and metastasis.

**Table 3:** Comparison of CTCs and ctDNA [30].

CTCs	ctDNA
**More steps for isolation than ctDNA**	Easier to isolate than CTCs
**Can be cultured and used for functional assays (both in vitro and in vivo)**	Cannot be cultured
**Can analyze DNA, RNA and protein**	DNA is more stable than RNA
**Sampling bias of captured cells (only cells of high affinity, size based)**	Small quantities of ctDNA in circulation
**Levels of CTCs can be used to predict response and resistance to therapy**	Levels of ctDNA can be used to predict response and resistance to therapy
**Heterogeneity can confound analysis**	Cell death under therapy could modify ctDNA levels
